# Recurrent Rash and Anemia: A Diagnostic Challenge of Angioimmunoblastic T-cell Lymphoma Mimicking Parvovirus B19 Infection

**DOI:** 10.7759/cureus.68517

**Published:** 2024-09-03

**Authors:** Taro Kunitomi, Taiju Miyagami, Yuji Kiyose, Hiroyuki Terukina, Ritsuko Kawabata, Yu Watanabe, Yusuke Yamamoto, Toshio Naito

**Affiliations:** 1 Department of Medicine, Faculty of Medicine, Juntendo University, Tokyo, JPN; 2 Department of General Medicine, Jyuntendo University Hospital, Tokyo, JPN; 3 Department of General Medicine, Faculty of Medicine, Juntendo University, Tokyo, JPN; 4 Department of Human Pathology, Faculty of Medicine, Juntendo University, Tokyo, JPN; 5 Department of General Medicine, Juntendo University, Tokyo, JPN

**Keywords:** anemia, fever, human parvo virus b19, urticarial-like rash, t-cell lymphoma

## Abstract

Angioimmunoblastic T-cell lymphoma (AITL) is a rare and challenging subtype of T-cell lymphoma often presenting with skin rashes and difficult diagnostic features. Its presentation can mimic other conditions, complicating accurate diagnosis. This case shows AITL in a 74-year-old man initially presenting with anemia that mimicked pure red cell anemia caused by parvovirus B19. The patient exhibited direct Coombs-positive anemia and recurrent urticarial-like rashes, which were initially misleading. This case emphasizes the critical need for considering lymphoma in patients presenting with direct Coombs-positive anemia and recurrent urticarial-like rashes It underscores the importance of revisiting and thoroughly assessing medical histories to enable accurate diagnosis, even when initial presentations suggest alternative diagnoses. Early recognition and appropriate management of AITL are crucial for improving patient outcomes.

## Introduction

Angioimmunoblastic T-cell lymphoma (AITL) is a rare and aggressive type of lymphoma, making up 1-2% of all non-Hodgkin's lymphomas [1]. In the 2016 World Health Organization(WHO) classification, AITL is grouped under nodal T-cell lymphomas with a follicular T-helper (TFH) phenotype [1]. AITL is relatively common among T-cell lymphomas, accounting for about 20% of cases in both Asian and Western populations [2, 3]. Most patients are diagnosed around the age of 69, and 79.5% of cases are in advanced stages when found [4].

The most common clinical symptom is swelling of the lymph nodes throughout the body, and other symptoms include anemia and skin rash [1, 5, 6]. Dermatological symptoms exhibit a variable presentation, ranging from erythematous eruptions to morbilliform rashes. The pathological findings produce a variety of findings, leading to the difficulty of diagnosis and poor prognosis [7]. Previous reports have shown that the 5-year survival rate is around 33-44% [1, 8, 9]. The skin rash can also vary, and it can sometimes be mistaken for several diseases, such as autoimmune diseases or drug rash [10, 11]. Erythematous lesions accounted for 86% of the elementary dermatologic lesions, and pruritus was reported in 48% of cases [11]. In this case, we report a case of AITL that mimicked parvovirus B19 infection based on the findings of anemia and skin rash.

## Case presentation

A 74-year-old man was referred to our hospital with a one-month history of persistent malaise, mysterious rash, and normocytic anemia. He began experiencing generalized malaise one month ago. Despite this discomfort, he pushed through, hoping it would pass. However, his symptoms lingered, promoting a visit to his local doctor. Then, blood samples showed hemoglobin (Hb) 11.9 g/dL and C-Reactive Protein 1.2 mg/dL, indicating anemia and inflammation. Two weeks prior, after being bitten by a wild cat and contact with their grandchildren who appeared healthy, he suddenly noticed an erythematous rash on the right side of his abdomen and extremities. He was concerned, sought further evaluation, and was referred to our department for a thorough examination of his symptoms. 

Upon arrival, physical examination confirmed the presence of an erythematous urticaria-like rash on the abdomen and extremities, which did not have pruritic or edematous regions. Additionally, the left axillary and cervical lymph node adenopathy was 3 cm in diameter, had no pain, was non-adherent to surrounding tissues, and exhibited elastic stiffness. His medical history showed that his performance status was normal. In addition, he did not experience night sweats but a weight loss of 6.7% in one month. Laboratory tests showed a Hb level of 7.8 g/dL, a positive result for parvovirus B19 IgM (Table 1). 

**Table 1:** Laboratory tests when admitted to hospital

Laboratory indicator	Result	Normal range
White Blood Cell (WBC)	5.6×10^9 /L	3.9-9.7×10^9 /L
Red Blood Cell (RBC)	2.8×10^12 /L	4.3-5.67×10^12 /L
Hemoglobin (Hb)	7.8 g/dL	13.4-17.1 g/dL
Mean Corpuscular Volume (MCV)	83.2 fL	84.2-99 fL
Mean Corpuscular Hemoglobin Concentration (MCHC)	33.5 g/dL	31.8-34.8 g/dL
Red cell distribution width (RDW)	12.20%	11.9-14.5%
Platelet (Plt)	336×10^9 /L	153-346×10^9 /L
Fe	108 μg/dL	80-170 μg/dL
Total Iron-Binding Capacity (TIBC)	260 μg/dL	290-390 μg/dL
Ferritin	687 ng/mL	30-400 ng/mL
haptoglobin	40 mg/dL	17-169 mg/dL
Aspartate Aminotransferase (AST)	13 U/L	5-37 U/L
Alanine Aminotransferase (ALT)	10 U/L	6-43 U/L
Lactate Dehydrogenase (LDH)	226 U/L	124-222 U/L
Gamma-Glutamyl Transpeptidase (γ-GTP)	16 U/L	0-75 U/L
Total-bilirubin	0.97 mg/dL	0.4-1.2 mg/dL
Blood Urea Nitrogen (BUN)	24 mg/dL	9-21 mg/dL
Creatinine	0.74 mg/dL	0.6-1 mg/dL
C-reactive protein (CRP)	1.75 mg/dL	< 0.3 mg/dL
Estimated Glomerular Filtration Rate (eGFR)	78.7	N/A
Parvovirus B19 IgM antibody	Positive	-
Direct coombs test	Positive	-

At the time of his coming to the hospital, CT imaging from the neck to the pelvis was performed. It showed multiple lymph node adenopathy and splenomegaly.

**Figure 1:**
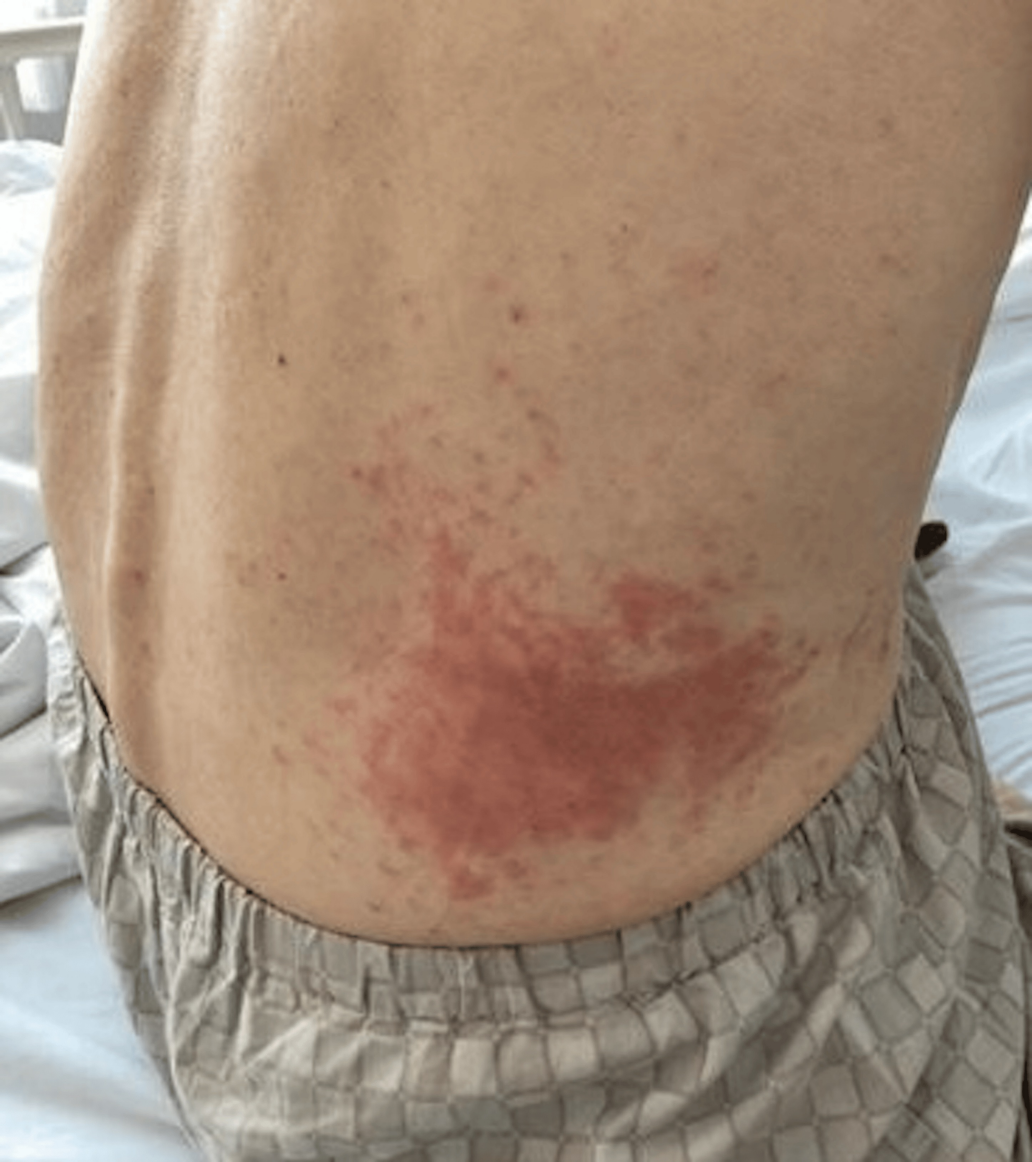
Rash during an exacerbation The skin lesions have merged and are getting worse. (arrow)

We initially suspected pure red cell aplasia (PRCA) caused by parvovirus B19 due to anemia with monocytopenia, a positive result for parvovirus B19, and the patient’s history of contact with children. Furthermore, the rash supported this suspicion. Therefore, assuming this, we decided to monitor the patient with symptomatic treatment. No treatment was offered to him, and the Hb level of the patient temporarily improved to 9.4 g/dL during the first five days. This suggested that we might be observing the recovery process of PRCA, and this would be the case with PRCA. However, the Hb level subsequently showed a declining trend of 7.8 g/dL on day 9. The skin lesions also changed during hospitalization. The erythematous rash was scattered over the trunk and extremities when he came to the hospital. On day 5, it widened, merged, and darkened in color, which looked like getting worse. However, on day 9, the color lightened, and the area shrank as if it was improving. Furthermore, it is difficult for us to understand what the direct Coombs test positive means. This could not be explained by PRCA, prompting a biopsy of axillary lymph nodes and skin lesions to differentiate diseases with recurrent skin rashes (Figure 1). 

**Figure 2:**
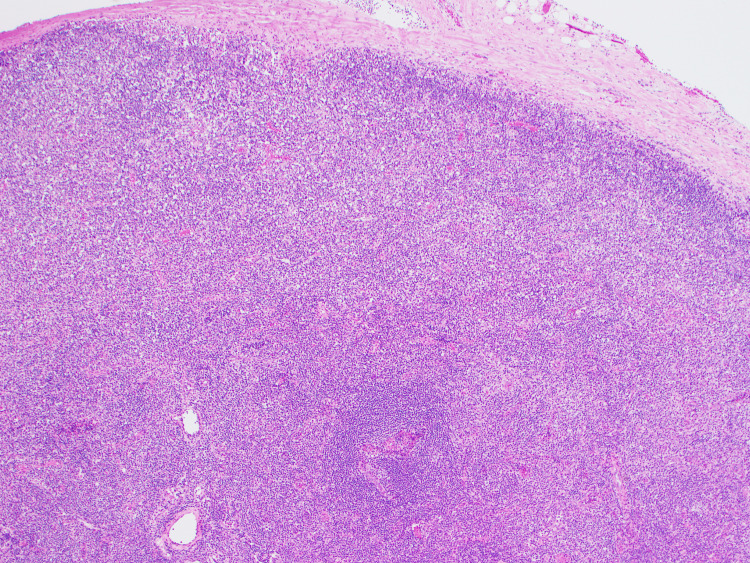
Pathology of the axillary lymph node under Hematoxylin and Eosin, 40x The biopsy from the left axillary lymph node. Note that the nodal structure is completely effaced accompanied by a diffuse polymorphic infiltrate and increased vascularity.

**Figure 3:**
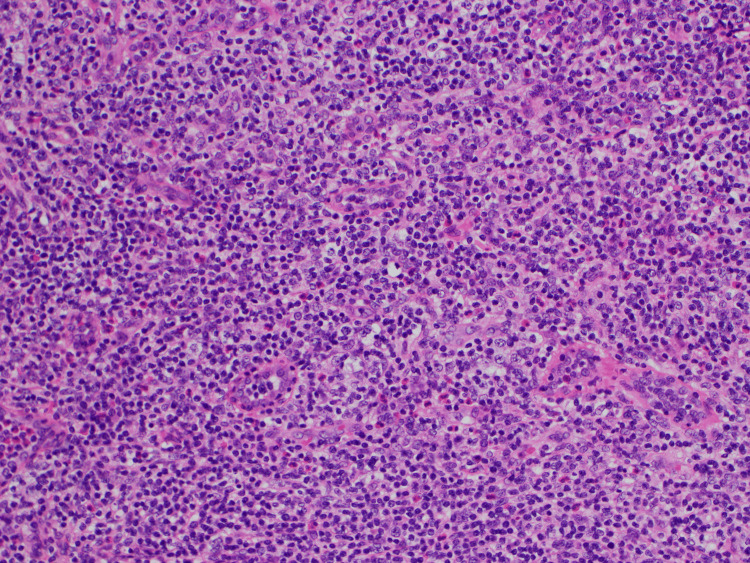
Pathology of the axillary lymph node under hematoxylin and eosin 200x The polymorphic infiltrate comprised atypical lymphocytes ranging from small to intermediate in size.

**Figure 4:**
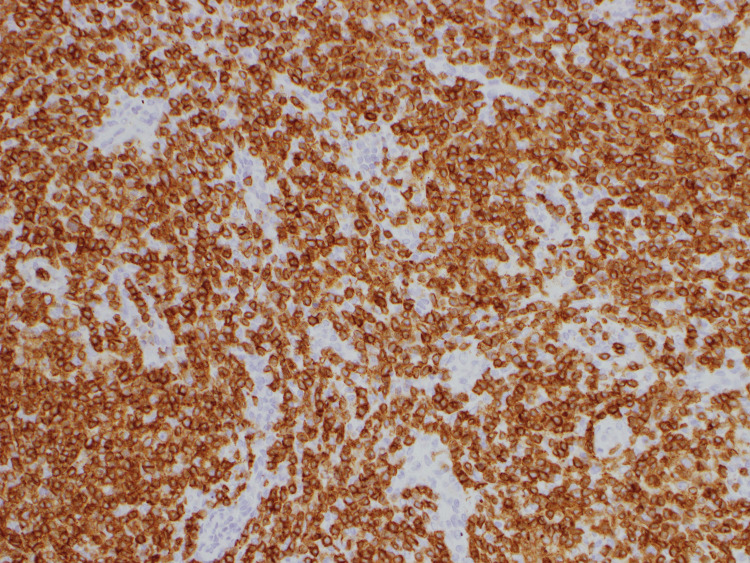
CD3-positivity by immunohistochemistry. Pathology of axillary lymph node under 200x magnification Atypical lymphocytes show positivity for respective markers.

**Figure 5:**
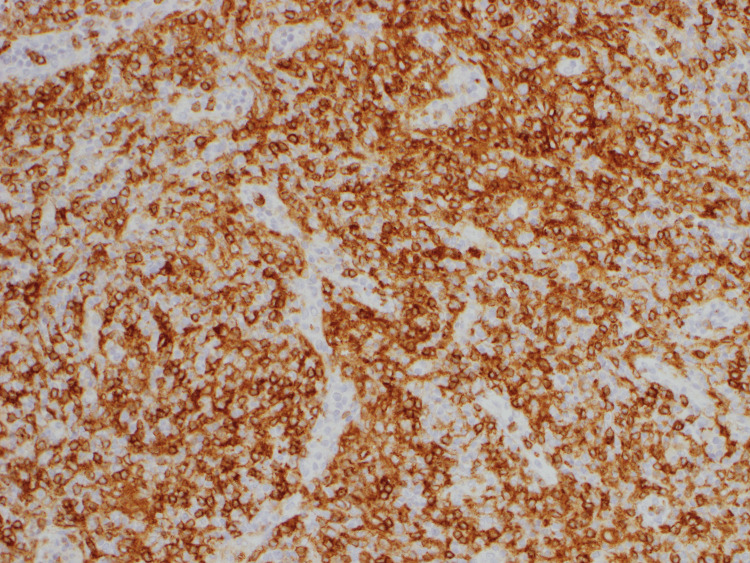
CD4-positivity by immunohistochemistry. Pathology of axillary lymph node under 200x magnification Atypical lymphocytes show positivity for respective markers.

**Figure 6:**
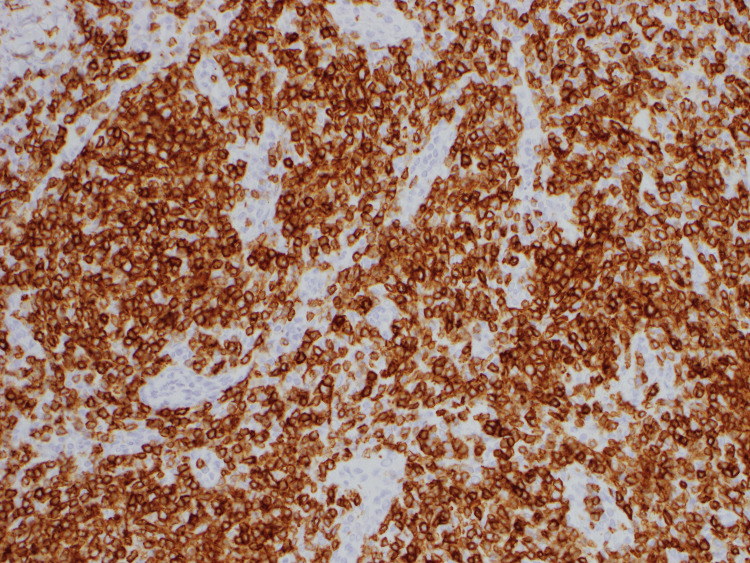
CD5-positivity by immunohistochemistry. Pathology of axillary lymph node under 200x magnification Atypical lymphocytes show positivity for respective markers.

**Figure 7:**
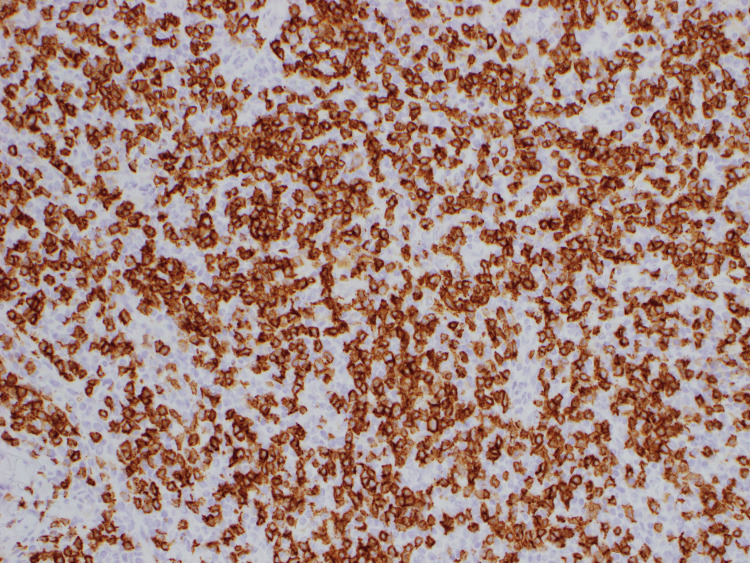
CD8-positivity by immunohistochemistry. Pathology of axillary lymph node under 200x magnification Atypical lymphocytes show positivity for respective markers.

**Figure 8:**
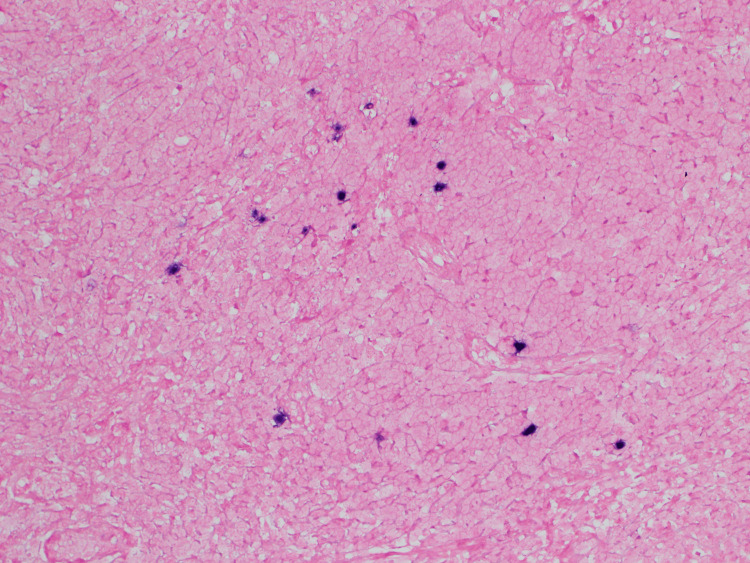
There is an admixture of cells positive for Epstein-Barr virus (EBV)-encoded small RNA in situ hybridization (EBER). Pathology of axillary lymph node under 200x magnification Epstein-Barr virus-encoded small RNA in situ hybridization (EBER-ISH) staining demonstrates the presence of EBV-encoded RNA in scattered cells in the node.

The lymph node biopsy revealed complete effacement of the nodal structure with a diffuse polymorphic infiltrate and increased vascularity. The infiltrate comprised atypical lymphocytes ranging from small to intermediate in size. Immunohistochemical analysis showed positivity for CD3, CD4, CD5, and CD8 markers in the atypical lymphocytes. Additionally, there were cells positive for Epstein-Barr virus-encoded small RNA in situ hybridization (EBER) (Figures 2-8). Skin biopsy showed the same markers in the lymphocytes. These four markers are not likely to appear in parvovirus B19. The histopathological findings were suggestive of AITL and confirmed by flow cytometry.

Considering the pathological feature of disrupted lymph node architecture, the diagnosis of angioimmunoblastic T-cell lymphoma(AITL) was made. This is classified as Ann Arbor Stage Ⅲ and has a score of 3 points on the International Prognostic Index. During hospitalization, the patient received two units of red blood cell transfusions due to a progressive decrease in Hb levels. Subsequently, considering ease of access, he was transferred to a different hospital. A-CHP therapy is carried out there, and the progress is good.

## Discussion

This case of AITL mimicked human parvovirus B19 infection. It initially presented with an erythematous skin rash following contact with healthy children. In immunocompetent patients, parvovirus B19 infection showed acute progress, whereas the symptoms appear chronically in immunocompromised patients. The typical skin rash associated with parvovirus B19 consists of erythematous lesions, consistent with this case's findings [12]. However, the recurrent nature of the rash, combined with changes in the Hb level, led us to reconsider the differential diagnosis. The skin lesions of AITL are seen in 49.0% of the patients and often present as multiple erythematous eruptions like this case [13]. Also, some collagen diseases like adult Still's disease and Sweet's disease should be considered [10]. However, these diseases show high fever and elevated inflammatory response and thus were excluded from this case. Also, recurrent symptoms are relatively common in lymphomas [14]. Therefore, recurrent urticaria-like rashes should prompt consideration of hematological diseases such as lymphoma, including AITL.

This case finally showed normocytic anemia with a positive result for the direct Coombs test. The rapid onset of anemia initially raised the possibility of pure red cell aplasia (PRCA). Infectious PRCA usually improves in immunocompetent patients within 2 weeks [15]. This was consistent with the present case, as hemoglobin levels improved during hospitalization. However, the positive direct Coombs test warranted discussion. Diseases positive for direct Coombs include autoimmune or drug-induced hemolytic anemias [1, 16]. False positives can occur with lymphoma, intravenous immune globulin administration, antiphospholipid syndrome, HIV, and malaria [15, 17]. Also, parvovirus B19 infection doesn't usually show a positive result. Although hemolytic anemia was suspected, it was unlikely as there were no changes in haptoglobin or LDH, reducing the possibility of Evans syndrome, a complication of AITL [18]. Re-evaluating positive and false-positive conditions, along with the lymph node adenopathy, led us to estimate that lymphoma was the most likely cause. Thus, following up on test results, including false positives, is essential when the clinical presentation does not match the suspected cause.

In this case, parvovirus IgM was positive. Parvovirus IgM has a sensitivity of 89.1% and specificity of 99.4%, making it a reliable test for definitive diagnosis [19]. The children who contacted the patient were not particularly ill, and the duration of the generalized malaise could not be explained solely by parvovirus. Epstein-Barr virus is known to be related to the progression of AITL, and some cases are reported [20]. However, there have been no reported cases of complications between parvovirus B19 and AITL. Therefore, the result may be coincidental. Additionally, we did not evaluate paired sera or polymerase chain reaction (PCR), so the actual situation is unknown. This case should be used as a learning experience for future cases.

## Conclusions

We encountered a case where AITL mimicked parvovirus. Recurrent progress of AITL can look like the recovery period of PRCA. Even if the patient has anemia, rash, and a positive result of parvovirus B19 IgM, we should not decide promptly that it is PRCA but broaden differentials, think about AITL, and consider the necessity of performing a biopsy. This is strongly true when there is data that cannot be explained just by PRCA, such as the positive result of the direct Coombs test. Furthermore, it is good to consider hemolytic anemia in cases with a positive direct Coombs test. In addition, when the other data does not fit the hemolytic anemia, it is essential to rule out false positives to verify the diagnosis. Additionally, this case combines AITL with parvovirus B19 infection. The relationship between these two things is unclear and worth researching further.
